# An Automated Modular Platform for Vascular Graft Assessment via Coronary-like Flow-Induced Stimulation

**DOI:** 10.3390/bioengineering13020221

**Published:** 2026-02-14

**Authors:** Elia Pederzani, Lucrezia Moro, Alessia Sofia Bolandrina, Sara Rega, Gianluca Lorenzo Perrucci, Gianfranco Beniamino Fiore, Monica Soncini

**Affiliations:** 1 Department of Electronics, Information and Bioengineering, Politecnico di Milano, 20133 Milan, Italylucrezia.moro@polimi.it (L.M.); alessiasofia.bolandrina@polimi.it (A.S.B.); gianfranco.fiore@polimi.it (G.B.F.); 2 Unit of Cardio-Oncology and Vascular Biology, Centro Cardiologico Monzino IRCCS, 20138 Milan, Italy; sara.rega@cardiologicomonzino.it (S.R.); gianluca.perrucci@cardiologicomonzino.it (G.L.P.)

**Keywords:** vascular engineering, automated culture platform, tissue-engineered vascular grafts (TEVGs), coronary artery bypass grafting (CABG), graft compliance characterization, coronary-like hemodynamics, biomechanical stimulation

## Abstract

Tissue-engineered vascular grafts (TEVGs) represent a promising alternative for coronary artery bypass grafting (CABG). However, replicating the mechanical and biological complexity of native vessels remains a major challenge. Compliance mismatch, local hemodynamics, and insufficient endothelialization are recognized as key contributors to maladaptive remodeling and graft failure. These limitations highlight the urgent need for advanced experimental platforms and standardized physical stimulation procedures to investigate these underlying biomechanisms and support the development of more effective TEVGs. In this work, we present an automated, modular platform designed to quantitatively characterize graft compliance and replicate coronary hemodynamics. The system integrates automated experimental procedures within a modular, incubator-compatible design, enabling an intuitive setup and real-time monitoring of physical parameters. Its modular architecture and dedicated control algorithms provide high adaptability, enabling its application across a broad range of experimental conditions. Bench testing demonstrates that the platform can automatically reproduce the pressure regimes defined by ISO standard and generate coronary-like flow-induced stimuli. These results confirm the innovative capability of the system to provide controlled and physiologically relevant conditions suitable for the investigation of key phenomena involved in CABG failure. In perspective, the platform offers a valuable tool for advanced mechanobiological studies in vascular tissue engineering.

## 1. Introduction

Coronary artery disease (CAD) remains a leading cause of mortality worldwide and arises from the progressive obstruction of coronary arteries by atherosclerotic plaque [1,2]. Although multiple therapeutic strategies exist—including pharmacological therapies, percutaneous coronary intervention and stenting—coronary artery bypass grafting (CABG) continues to be the preferred intervention for high-risk patients with complex lesion patterns and/or unfavorable clinical histories [3]. CABG restores myocardial perfusion by anastomosing autologous or synthetic conduits to stenotic coronary arteries to bypass atherosclerotic occlusions. Saphenous vein grafts remain the most widely used conduits due to their accessibility, handling characteristics, and sufficient length for multi-vessel bypass [3,4,5]. Nevertheless, the long-term success of venous grafts is compromised by intimal hyperplasia-driven luminal narrowing, resulting in 10–25% graft occlusion within the first year [1,6,7,8,9]. Furthermore, autologous vessels are not always available due to prior harvest, underlying vascular disease, or patient-specific anatomical constraints.

This clinical limitation has spurred considerable efforts in the field of bioengineering and material science to develop alternative vascular grafts that can replicate the structural, mechanical, and biological functions of native vessels [10,11]. Traditional synthetic grafts (e.g., polyethylene terephthalate (PET) and expanded polytetrafluoroethylene (ePTFE)) are a useful alternative for large-diameter vessels; however, they have shown limited success in small-diameter applications (Ø < 5 mm) [11,12,13]. Indeed, small-diameter vascular grafts (SDVGs) remain particularly prone to failure mainly as a result of intimal hyperplasia (IH), thrombosis and infection, which significantly limits their clinical applicability in coronary bypass procedures [14,15]. Tissue-engineered vascular grafts (TEVGs) represent a promising strategy for overcoming these limitations and serving as alternative conduit vessels for clinical use. However, replicating the mechanical and biological properties of native tissues remains challenging due to their complex structure and composition, and numerous aspects still require further improvements to ensure their safe and reliable application [16,17]. Consequently, the development of novel TEVGs for small-caliber vessel replacement has become one of the most intensively investigated and technically demanding areas in vascular tissue engineering [18]. The prevailing hypothesis suggests that a lack of luminal endothelialization, local hemodynamics and compliance mismatch between the graft and host artery are among the leading contributors to maladaptive vascular remodeling and subsequent graft failure [6,7,14,15,18,19,20,21,22,23]. Despite extensive investigations of the interplay between these factors, a comprehensive and unified understanding of the underlying mechanobiological phenomena remains elusive. Unraveling these pathophysiological phenomena continues to pose significant challenges, underscoring the urgent need for effective experimental models that can faithfully mimic in vivo conditions [24]. A physiologically relevant model that accurately replicates coronary-specific hemodynamics would enable an in-depth investigation of the biomechanisms underlying CABG failure, thereby supporting the development of emerging vascular graft technologies.

In this scenario, advanced in vitro culture systems constitute an effective approach for investigating vascular remodeling phenomena, enabling a precise control over applied stimuli and reproducible cell seeding procedures [25,26]. The fine-tuned control of biomechanical cues is particularly critical in the development of small-diameter TEVGs for CABG, given that coronary arteries are subjected to flow-induced stimuli that significantly differ from those in the systemic circulation. The coronary circulation is governed by unique counter-phase hemodynamics: systolic contraction induces an increase in coronary resistance, reducing blood flow during peak pressure, whereas diastolic relaxation drives dominant perfusion at lower pressure [27,28,29,30,31]. This counter-phase relationship between pressure and flow generates physical and mechanobiological conditions (i.e., peculiar wall shear stress patterns, oscillatory components, and temporal gradients), critically regulating endothelial behavior and vascular remodeling [32,33,34,35].

In the literature, beyond numerous culture systems capable of hosting tubular samples and applying single or combined systemic-like flow-induced stimuli [36,37,38,39,40,41,42,43,44,45], various platforms have been developed to replicate coronary hemodynamics [46,47,48,49,50,51,52]. However, most of these systems are poorly aligned with standard biological procedures: their complexity and large footprint prevent their placement within standard incubators, they offer limited sample accessibility, inadequate throughput for longitudinal studies, and do not support efficient and reproducible cell seeding procedures. Furthermore, their low degree of automation demands extensive user intervention and technically complex maneuvers to replicate the fluid dynamics of coronary circulation, thereby reducing operational precision and compromising the reliability and reproducibility of experimental outcomes. These limitations underscore the urgent need for a physiologically relevant coronary hemodynamic simulator capable of supporting the assessment of graft mechanical properties and enabling the investigation of fluid dynamic stimuli in relation to vascular cell behavior and remodeling.

To address this critical gap, we developed a culture system designed to perform automated cell seeding and apply controlled flow-induced stimuli to tubular samples. The system is fully compatible with conventional biological procedures, including sterilization protocols, assembly under a biological safety cabinet, and placement within standard incubators. Its effectiveness has been demonstrated in biological experiments involving 3D cell co-cultures and flow-induced stimulations [53].

Building on this groundwork, here we present the development of a modular, automated platform engineered to quantitatively characterize graft compliance—a parameter widely recognized as critical in vascular remodeling due to the mismatch between graft and native vessels—and to replicate coronary-specific hemodynamic conditions. The platform serves as a valuable tool to define rigorous physical stimulation procedures aimed at investigating key biomechanical phenomena involved in vascular remodeling and failure in CABG. Its core functionalities are enabled through the integration of two dedicated culture chambers, along with the implementation of programmable control algorithms for automated operation. Specifically, one chamber is designed for the mechanical characterization of tubular samples in accordance with the ISO 7198 standard [54], while the second is intended for advanced longitudinal biological studies, supporting reproducible automated cell seeding and faithfully replicating the peculiar counter-phase hemodynamics of the coronary circulation. These technological features make the platform a novel, incubator-compatible device characterized by high adaptability, intuitive system setup, and real-time monitoring of experimental parameters, while enabling the controlled generation of physiologically relevant flow-induced stimuli. Bench testing of both system configurations demonstrates the platform’s ability to assess the compliance of tubular samples and to replicate diastolic-dominant perfusion and systolic flow attenuation, thus establishing its effectiveness as a versatile culture system for perspective mechanobiological investigations in vascular tissue engineering.

## 2. Materials and Methods

### 2.1. Modular Culture Platform for Vascular Engineering

**Figure 1:**
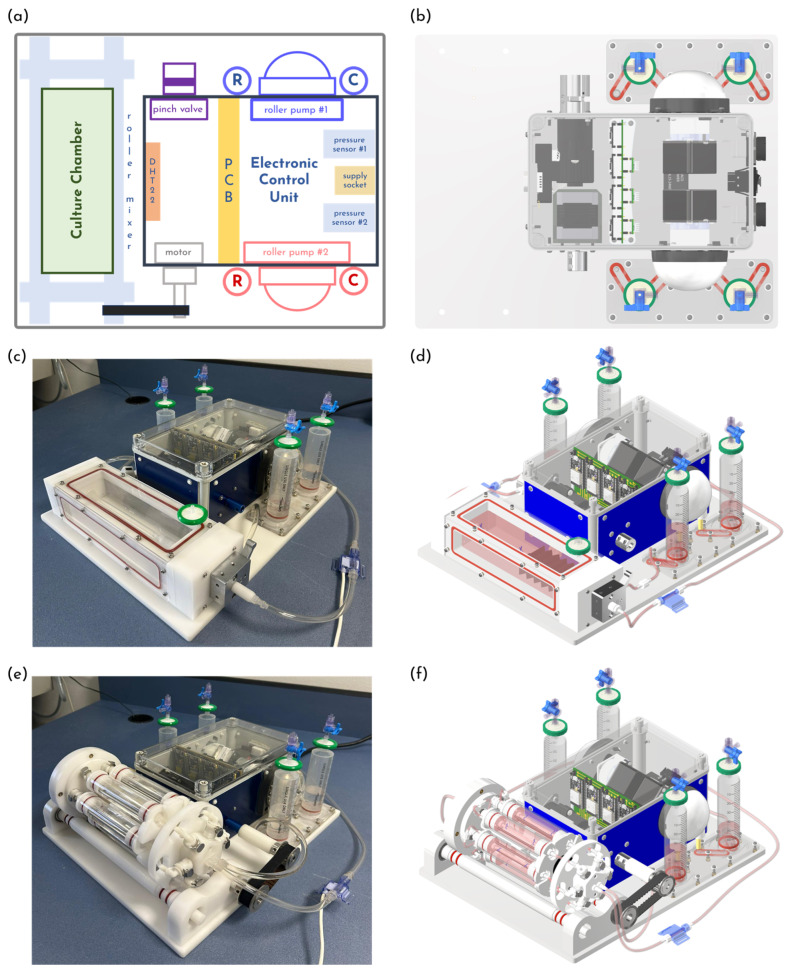
(**a**) Modular system design scheme (“R”, reservoir; “C”, compliance; “P”, pressure sensor; “PCB”, printed circuit board). (**b**) Culture platform CAD model (top view). Modular platform combined with (**c**,**d**) the graft characterization chamber and (**e**,**f**) the multi-sample chamber.

**Figure 2:**
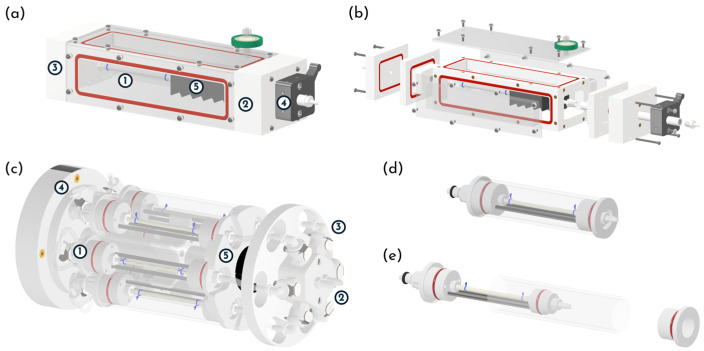
(**a**,**b**) Graft characterization chamber assembly and exploded view: ① sample; ②, ③ lateral panels; ④ shaft locking element; ⑤ bellow; (**b**) graft characterization chamber exploded view. (**c**) Multi-sample chamber: ① culture unit, ② inlet/outlet of the hydraulic circuits, ③ valved connector, ④ extraluminal luer-lock connectors, ⑤ locking element; (**d**,**e**) culture unit assembly and exploded view.

The culture platform is designed to enable the assessment of graft mechanical properties and to support longitudinal biological experiments, allowing automated cell seeding and in vivo-like flow-induced stimulation. Its design concept relies on a modular architecture with an electronic control unit managing the electromechanical peripherals and allowing the flexible integration of different culture chambers (Figure 1 and Figure 2). This approach provides a high degree of adaptability, making the platform versatile and suitable for a wide range of experimental scenarios. All components are mounted on a polyoxymethylene (POM) base, making the system compact, easily transportable, and fully compatible with a standard incubator. The system is managed through a graphical user interface (GUI), enabling intuitive monitoring and control of experimental parameters via a wireless communication protocol.

#### 2.1.1. Electronic Control Unit

The electronic control unit consists of an IP68 enclosure housing four electromechanical peripheral devices—a stepper motor, two peristaltic pumps and a proportional pinch-valve—and a custom-designed printed circuit board (PCB) integrating four ESP32^®^ microcontrollers (Espressif Systems, Shanghai, China) (Figure 1a).

**Figure A1:**
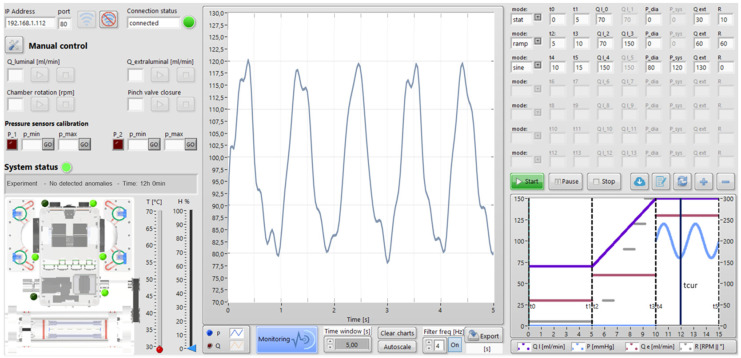
Graphic user interface for wireless control: left side for direct platform operation (“Manual control”, “Pressure sensors calibration”, “System Status”), central panel for real-time pressure and flow rate monitoring and data export, and right side for protocol parameter settings and experiment management.

The ESP32^®^ microcontrollers—mounted on the PCB and programmed in a main–subordinate configuration—manage all operational tasks of the culture system. The microcontrollers receive user-defined commands via a custom wireless graphic user interface (GUI) developed in LabVIEW^®^ 2023 (National Instruments, Austin, TX, USA) (Figure A1), communicating through the TCP/IP protocol to ensure stable and responsive remote control. The GUI allows either a manual operation of each peripheral or a pre-programmed experimental execution, including user-defined parameters such as duration, flow rate, pressure profile, and culture chamber rotation (further details are reported in section “Appendix A.1. Graphic User Interface for Wireless Control” of Appendix A). The microcontrollers continuously monitor key system parameters—temperature and humidity inside the control unit, chamber rotation, and pressure values—and transmit them to the GUI for real-time visualization. Furthermore, if any measured values exceed predefined thresholds, the microcontrollers interrupt the functioning of the peripherals, thereby ensuring safe experimental conditions.

Two independent hydraulic circuits are driven by WP1100 roller pumps (Welco Co., Ltd., Tokyo, Japan) (maximum flow rate: 600 mL/min). Each circuit incorporates a reservoir and a filtering element designed to dampen pump-induced pulsatility, both realized using sterile, disposable 60 mL syringes secured via bayonet locking on a dedicated mounting stage. Each stage is composed of three separable PMMA plates, allowing effective decontamination and sterilization. Fluidic connections are established through barbed connectors, luer-lock connectors and oxygen-permeable Tygon^®^ 3350 silicone tubing (Saint-Gobain, Charny-Orée-de-Puisaye, France). Syringe mounting, tubing connection to the selected culture chamber and other components, and circuit filling are performed by the operator through simple manual operations. The hydraulic circuits are dimensioned to allow for gas exchange—particularly oxygen diffusion required for the maintenance of cell viability—once the system is placed inside a standard incubator.

A stepper motor (17HS13-0404S, Sanyo Denki Co., Ltd., Tokyo, Japan)) drives a roller mixer apparatus, enabling programmable control of the chamber angular position or continuous revolution speed to implement cell seeding protocols.

**Figure 3:**
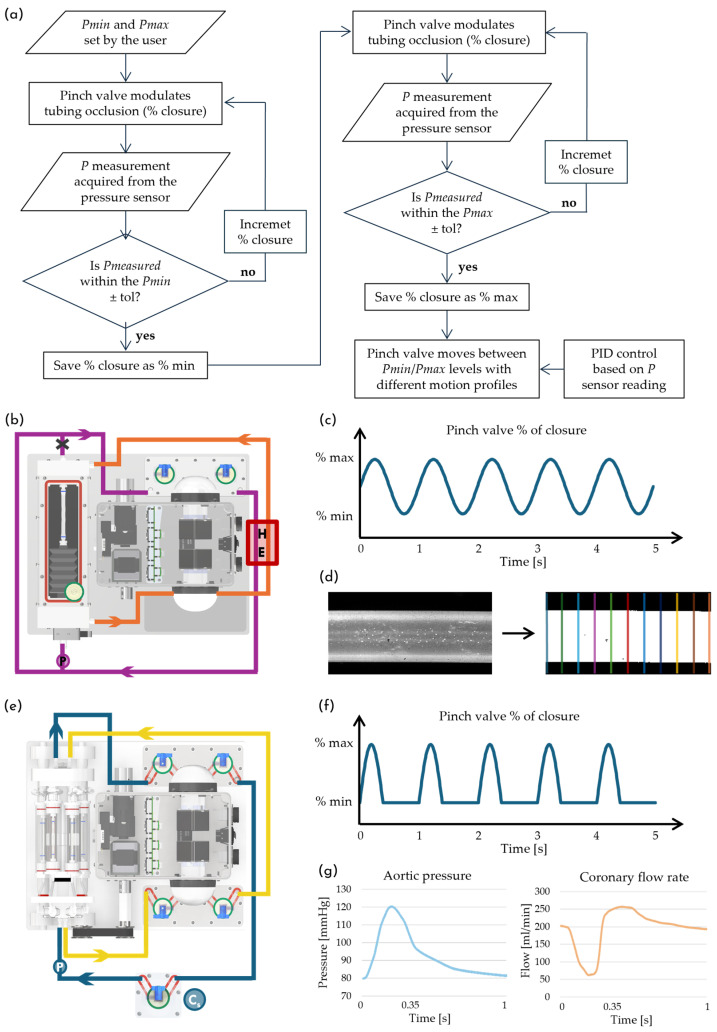
(**a**) Workflow of the pinch-valve control algorithm. (**b**) Top view of the platform with the graft characterization chamber for compliance assessment; purple line: hydraulic circuit for controlled sample pressurization; orange line: extraluminal fluid recirculation; “HE”: heat exchanger; “P”: pressure sensor. (**c**) Pinch-valve closure profile to generate pulsatile pressure during compliance testing. (**d**) Image analysis workflow for quantifying diameter deformation of tubular samples. (**e**) Top view of the platform with the multi-sample chamber for coronary-like stimulation; blue line: hydraulic circuit for coronary-like stimulation; yellow line: circuit for steady-flow control samples; “Cs”: service compliance chamber; “P”: pressure sensor. (**f**) Pinch-valve closure profile used to replicate coronary-like flow-induced stimuli. (**g**) Reference physiological aortic pressure and left coronary artery flow waveforms (redrawn starting from published data [58,59,60,61,62,63]).

The platform automatically achieves the desired pressure regime by adjusting the compression of the silicone tubing via a proportional pinch-valve (ASCO^TM^ S170XA01X2900VU, Emerson, St. Louis, MO, USA), based on real-time pressure measurements. The user can define two pressure levels, $P_{min}$ and $P_{max}$, through the GUI, which are then dynamically applied to the tubular sample. This functionality adds a further level of versatility and ease-of-use, as it eliminates the need for complex manual adjustments and preserves sterility, since none of the pinch-valve components are in contact with the fluid. The pinch-valve control algorithm operates in two stages: an initial pre-setting phase to determine the occlusion levels required to reach the target pressures, followed by the controlled dynamic stimulation phase (Figure 3a). Specifically, during the pre-setting phase, the pinch-valve gradually adjusts its closure level while pressure is continuously measured by a sensor (PREPS-N-000, PendoTECH^®^, Plainsboro, NJ, USA) and compared to the user-defined target value. When the measured pressure value falls within the specified tolerance around $P_{min}$, the corresponding pinch-valve closure is set. The same procedure is then repeated to identify the closure level associated with $P_{max}$. Once the pinch-valve closure levels corresponding to $P_{min}$ and $P_{max}$ have been established, the pinch-valve can reproduce different user-defined pressure waveforms. During this stimulation phase, a closed-loop proportional–integral–derivative (PID) controller continuously modulates the pinch-valve closure levels to maintain the desired pressure regime, ensuring consistent mechanical stimulation of the sample. This innovative approach is employed in both compliance assessment and coronary-like flow-induced stimulation setups by simply modifying the pinch-valve motion profile. This further highlights the platform ease-of-use and the versatility of the defined physical stimulation procedures.

#### 2.1.2. Culture Chambers

The culture platform is designed following a modular approach to ensure adaptability, enabling the integration of the chamber dedicated to vascular graft characterization or, alternatively, the multi-sample chamber for longitudinal biological studies.

The graft characterization chamber is directly mounted onto the culture platform (Figure 1c,d). It consists of three POM elements—a main frame (inner dimensions: 188 mm × 50 mm × 50 mm) and two lateral panels (each composed of two separate components)—with all components fastened by screws and hydraulically sealed at each interface using O-rings (Figure 2a,b). Three transparent PMMA plates are fastened to the main frame to provide optical access. The top plate is removable, allowing the operator to access the sample at any time. All chamber components can be fully disassembled, facilitating straightforward decontamination and autoclave sterilization (except for the PMMA plates). The tubular sample is mounted using two barbed connectors—one screwed into the lateral panel and the other into a sliding shaft—allowing the accommodation of diameters ranging from 1 to 8 mm. Sample length (10–160 mm) is set by means of the sliding shaft, which can be locked at the desired position using a shaft locking external mechanism (Figure 2a, element ④). To minimize the priming volume, the chamber incorporates a flexible bellows (Figure 2a, element ⑤) connected between the sliding shaft end and the lateral panel. The lateral panels integrate branched channels that ensure uniform adventitial recirculation through the hydraulic circuit that is connected to the chamber via luer-lock fittings.

To enable advanced longitudinal biological studies in vascular tissue engineering field, we develop a multi-sample chamber compatible with the previously validated rotational cell seeding protocol [53]. It is placed on a roller mixer rigidly secured to the culture platform and coupled to the stepper-motor shaft via a belt–pulley drive, enabling automated rotational cell seeding (Figure 1e,f). The multi-sample chamber (Figure 2c) consists of a revolver-like structure that accommodates up to six culture units (Figure 2d), each hosting a tubular sample. The revolver-like structure is equipped with two inlets and two outlets (Figure 2c, element ②); each inlet branches into three internal channels, allowing the perfusion of up to three culture units with the same fluid. The channel geometry is designed to ensure uniform flow distribution among the three culture units per inlet. This configuration supports two parallel experimental triplicates, which is particularly advantageous for longitudinal studies requiring sequential sample retrieval and analysis at multiple time points. Each culture unit comprises three components: a sample housing, a PMMA tube (length 113 mm, inner diameter 24 mm, wall thickness 2 mm), and a locking cap (Figure 2d,e). By swapping the sample-mounting threaded connectors, each culture unit can house tubular specimens across a wide range of sizes (diameter: 1–8 mm; length: 10–80 mm), thus improving the overall multi-sample chamber adaptability. The luminal connection of each culture unit to the revolver-like structure is achieved via valved connectors, which allow individual culture units to be mounted or removed without compromising the hydraulic integrity of the remaining system (Figure 2c, element ③). The extraluminal compartment of each culture unit can be manually filled or exchanged through luer-lock connectors (Figure 2c, element ④). Both the revolver-like structure and the culture units can be fully disassembled, enabling rapid decontamination and steam sterilization through autoclaving (except for the PMMA tubes).

### 2.2. Procedure for Vascular Graft Compliance Characterization

Dynamic radial compliance is a critical biomechanical property of vascular grafts. Matching the compliance of the graft to that of the native vessel is essential to minimize flow disturbances and reduce the risk of maladaptive vascular remodeling [55]. For these reasons, compliance quantitative assessment represents a necessary first step toward the investigation of biomechanical phenomena involved in vascular remodeling and failure in CABG.

To address this, we develop an automated protocol for the measurement of this key functional parameter, in accordance with the ISO 7198 standard [54]. The protocol is implemented using the culture platform equipped with the graft characterization chamber (Figure 2a) and set up as illustrated in Figure 3b. According to the standard, tubular samples must be pressurized at 37 °C under three distinct pulsatile pressure regimes (50–90 mmHg, 80–120 mmHg, and 110–150 mmHg) at a frequency of 1 Hz. To achieve these conditions, the pinch-valve closure is dynamically modulated via the previously described algorithm (Figure 3a), enabling a precise control of the luminal pressure within the sample. The imposed motion law controlling the pinch-valve follows a sinusoidal profile (Figure 3c). Pressure measurements are acquired using a PREPS-N-000 sensor (PendoTECH^®^, Plainsboro, NJ, USA) at 40 Hz and then filtered using a 3-point moving average prior to the analysis.

For this functional characterization, one hydraulic circuit is dedicated to the controlled pressurization of the sample (purple line, Figure 3b), while the second circuit is used to recirculate the extraluminal fluid, ensuring homogeneous temperature distribution (orange line, Figure 3b). Both luminal and extraluminal fluids are maintained at 37 °C by means of a custom heat exchanger consisting of a copper coil immersed in a thermostatic reservoir (“HE” element, Figure 3b).

During the controlled pulsatile pressurization of the sample, the platform is positioned under a stereo microscope (SMZ1000, Nikon Instruments Inc., Tokyo, Japan) equipped with a high-speed camera (Miro Vision 2, Phantom, San Francisco, CA, USA) for image acquisition (time window 30 s at 150 fps). Images are analyzed using a custom MATLAB R2025b script (MathWorks, Inc., Natick, MA, USA), which first registers and binarizes each frame and subsequently computes the outer diameter of the graft. The diameter is obtained by counting the number of white pixels along the vertical direction; this measurement is repeated at ten evenly spaced positions along the longitudinal axis, and the resulting values are averaged (Figure 3d). For each frame, the script extracts the minimum ($D_{P_{min}}$) and maximum ($D_{P_{max}}$) diameters, subtracts the wall thickness in accordance with standard ISO 7198 [54], and calculates the dynamic radial compliance (C, % 10^−2^ mmHg^−1^) according to Equation (1):

(1) $$C=\frac{(D_{P_{max}}-D_{P_{min}})/D_{P_{min}}}{P_{max}-P_{min}}\cdot 10^{4}$$

where $P_{max}$ and $P_{min}$ are the maximum and minimum of the pressure range of interest in mmHg, and $D_{P_{max}}$ and $D_{P_{min}}$ are the related inner diameters in mm.

A silicone tubular phantom (inner diameter 3.2 mm and wall thickness 0.4 mm), dimensionally comparable to small-caliber vascular grafts [56,57], is used to validate the implemented procedure and the performance of the platform with the dedicated culture chamber. The silicone phantom is mounted onto barbed connectors within the culture chamber, secured using vessel loops and pre-tensioned by adjusting the position of the sliding shaft, in accordance with standard ISO 7198 [54].

### 2.3. Coronary-like Flow-Induced Stimulation

To investigate the mechanobiology of TEVG remodeling in vitro under a realistic CABG scenario, it is essential to ensure proper cellularization of the tubular construct and to replicate the hemodynamics of the coronary circulation, specifically its characteristic counter-phase pattern. To obtain a coronary in vivo-like simulator, we employed the culture platform equipped with the multi-sample chamber (Figure 2c) and we designed an ad hoc hydraulic circuit to replicate diastolic-dominant perfusion. This configuration enables longitudinal studies and automated cell seeding.

The platform is tested over 14 consecutive days to assess both the generation and the long-term maintenance of the desired fluid dynamic conditions. The system is placed inside a standard cell culture incubator to closely simulate the configuration intended for biological experiments and to confirm its compatibility with the incubator in terms of footprint, handling, and heat dissipation. Six tubular silicone phantoms—identical to those employed in the compliance assessment and dimensionally representative of small-caliber vascular grafts—are mounted within the multi-sample chamber and secured to the barbed connectors using vessel loops.

Three of the six tubular samples are subjected to coronary-like flow-induced stimulation, perfused in parallel using one roller pump and its related reservoir/filtering stage (blue line, Figure 3e). To generate a diastolic-dominant perfusion profile with systolic flow attenuation, the proportional pinch-valve is programmed to cyclically compress the silicone tubing located between the outlet of the samples and the reservoir; additionally, a service compliance chamber is placed upstream of the samples (Figure 3e, element “C_s_”). When the pinch-valve increases compression, the hydraulic circuit resistance rises, leading to an increased pressure and reduced flow rate, as part of the fluid volume is temporarily stored in the service compliance. Conversely, when the pinch valve relaxes tubing compression, the resistance decreases and circuit pressure drops, while the flow rate transiently exceeds the average value due to the release of fluid from the service compliance. Automated control of the pinch-valve is critical for achieving consistent and tunable flow-induced stimulation. As previously described, the pinch-valve percentage of closure is regulated in real time through a feedback loop control algorithm (Figure 3a) based on a pressure sensor located upstream of the samples (Figure 3e, element “P”). The motion profile of the pinch-valve followed a piecewise-defined function: during the systolic phase, the pinch-valve performed a half-period sinusoidal closure over 0.35 s, followed by a 0.65 s diastolic phase in which the pinch-valve remained at its minimal compression level (Figure 3f). Figure 3g shows the pressure and flow waveforms of the left coronary artery considered physiological and representative of a healthy adult male [58,59,60,61,62,63]. These profiles are used as a reference for replicating coronary-like hemodynamics through the developed experimental setup. The remaining three samples are perfused under steady-flow conditions, serving as a control group. They are perfused in parallel through the second roller pump, with its pulsatility attenuated by their reservoir/filtering stage (yellow line, Figure 3e).

To mimic the viscosity of human blood, distilled water is supplemented with 50% (*w*/*w*) glycerol, resulting in a dynamic viscosity of approximately 3.5 mPa·s at 37 °C. The roller pumps in both hydraulic circuits are set to a flow rate of 540 mL/min, yielding a mean perfusion rate of 180 mL/min per sample, consistent with physiological values reported for the left coronary artery [58,59,60]. Pressure and flow rate are recorded daily for 14 days for one of the three coronary-like stimulated samples. Pressure data are acquired at 40 Hz using a PREPS-N-000 sensor (PendoTECH^®^, Plainsboro, NJ, USA), while flow rate data are acquired at 200 Hz using an ultrasound flowmeter HT110R equipped with a 1/8” probe (Transonic System Inc., Ithaca, NY, USA); both signals are filtered using a 3-point moving average prior to analysis.

## 3. Results

The platform is bench tested to evaluate its capability to support the defined physical procedures for assessing graft compliance and replicating physiologically relevant coronary-specific conditions. Particularly, we focus on its key feature of automatically generating controlled fluid-dynamic conditions through pinch-valve modulation. In the following sections, we report the results of compliance characterization and the evaluation of the coronary-like flow-induced stimulation on silicone phantoms.

### 3.1. Assessment of the Culture Platform for Compliance Characterization

**Figure 4:**
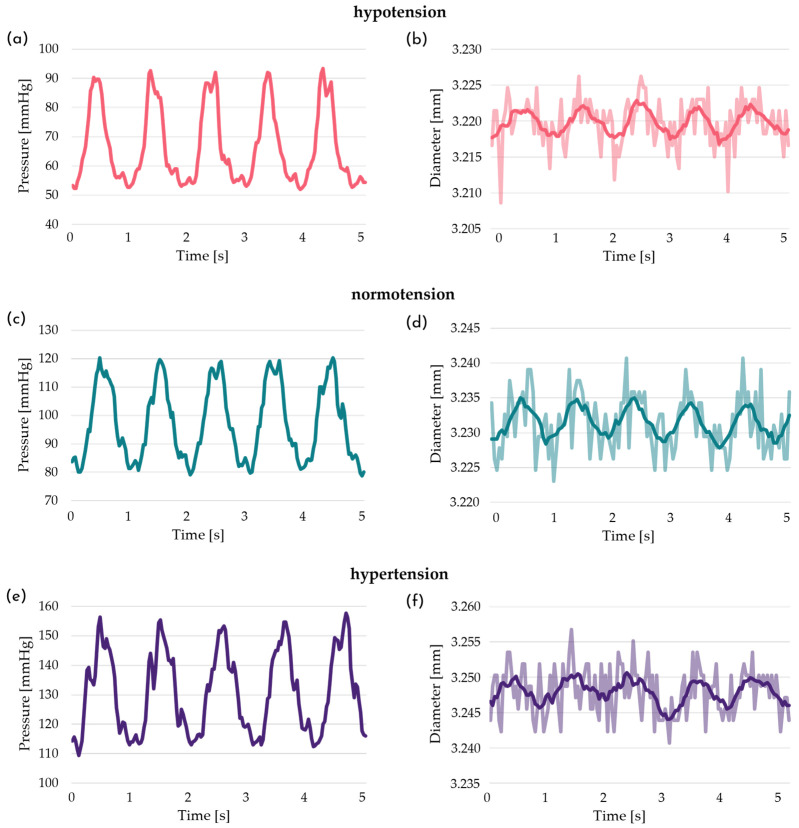
Dynamic pressure stimulation and corresponding diameter deformation of the silicone phantom under (**a**,**b**) hypo-, (**c**,**d**) normo-, and (**e**,**f**) hypertension condition. (**a**,**c**,**e**) Imposed pressure waveform over five periods and (**b**,**d**,**f**) associated cyclic diameter variations (raw data in a lighter color, filtered data profile in a darker color).

The dynamic radial compliance is evaluated on the silicone phantom in accordance with ISO 7198 [54]. The platform successfully reproduced the three pressure regimes defined by the standard. The acquired pressure–diameter data used to calculate compliance under hypo-, normo-, and hypertensive conditions are qualitatively reported: Figure 4a,c,e show the generated pressure profiles, while Figure 4b,d,f exhibit the cyclic diameter variation in the silicone phantom in response to the applied pressurization.

Under all three conditions, the average values—calculated over thirty consecutive periods (1 s per period)—for minimum and maximum pressures, and consequently the associated pressure variation, are consistent with the standard ISO 7198 [54], also exhibiting an acceptable standard deviation (Table 1). By correlating the sample diameter variation with the applied pulsatile pressurization, the dynamic radial compliance is quantified under hypo-, normo-, and hypertension conditions (Table 1).

### 3.2. Assessment of the Culture Platform for Coronary-like Stimulation

**Figure 5:**
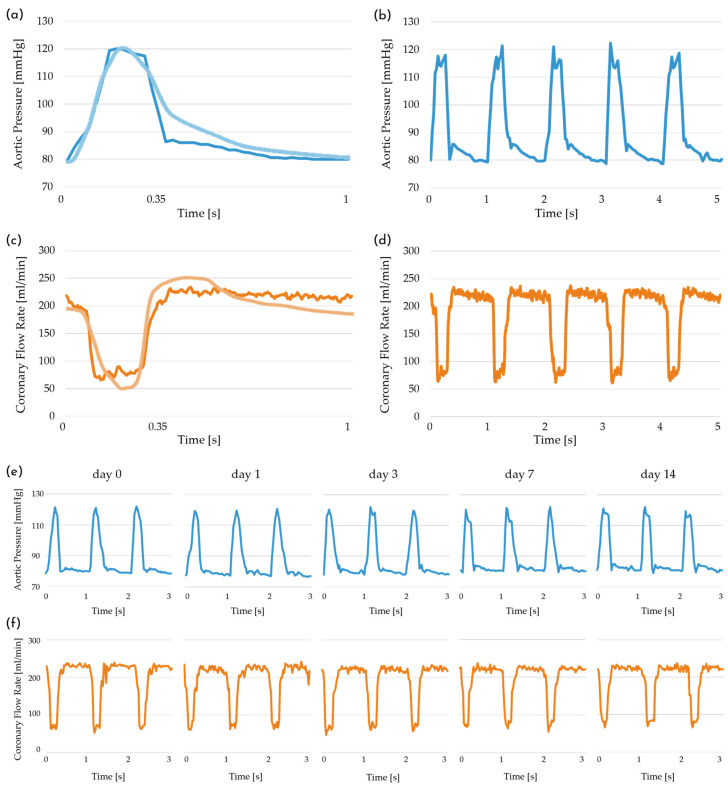
(**a**) Pressure measured at the sample inlet (blue line) compared with the physiological target pressure (light blue line; redrawn starting from published data [58,59,60,61,62,63]); (**b**) pressure acquired over five consecutive periods. (**c**) Flow rate measured at the sample inlet (orange line) compared with the physiological left coronary flow rate (light orange line; redrawn starting from published data [58,59,60,61,62,63]); (**d**) flow rate acquired over five consecutive periods. (**e**,**f**) Pressure and flow rate acquired during a long-term platform assessment at day 0, day, day 3, day 7, and day 14.

Coronary-like stimulation is performed on the silicone phantoms to assess the ability of the culture platform to reproduce coronary-specific conditions. The acquired flow rate and pressure regime are compared with physiological profiles reported in the literature for left coronary hemodynamic conditions (Figure 5a,c) [58,59,60,61,62,63]. The generated waveforms are qualitatively analyzed by visualizing five periods (1 s per period) and quantitatively evaluated by calculating the mean and standard deviation of the pressure and flow rate data over a thirty-period interval (Figure 5b,d). In this experimental configuration, the platform successfully replicates the target coronary-like behavior, characterized by a diastolic-dominant flow and peculiar counter-phase between pressure and flow rate. Moreover, it maintains this peculiar fluid dynamics over time: pressure varies between 79.15 ± 0.62 mmHg and 119.98 ± 1.85 mmHg, with a mean excursion of 40.83 ± 1.97 mmHg; the flow rate has a mean value of 188.42 ± 2.09 mL/min (corresponding to an estimated wall shear stress of approximately 3.4 Pa [64]), with an average variation between minimum and maximum values of 165.81 mL/min. The obtained waveforms exhibit a close likeness to the physiological coronary profiles, particularly regarding the diastolic-dominant perfusion and systolic flow attenuation.

Over the 14-day long-term assessment, the pressure and flow rate consistently preserve their characteristic waveforms and counter-phase behavior (Figure 5e,f). Quantitative analysis of the acquired data, in particular minimum and maximum pressures, pressure variation, mean flow rate, and flow rate excursion show no significant variability throughout the 14-day period (Table 2).

Overall, these outcomes indicate that the feedback loop pinch-valve actuation, combined with PID control, enables the platform to automatically reproduce coronary-like flow-induced stimuli and to maintain them over long-term operations.

## 4. Discussion

In this study, we develop and test a modular, automated platform for quantitative compliance assessment and for coronary-like flow-induced stimulation. System functionality and effectiveness are evaluated in both settings, demonstrating the platform’s ability to automatically generate specific flow-induced stimuli through programmable control algorithms.

The platform equipped with the graft characterization chamber successfully applies the three pressure regimes required by ISO 7198 [54], while simultaneously acquiring real-time diameter variations (Figure 4) to assess graft compliance. This platform configuration represents a technological advancement over state-of-the-art systems, particularly in terms of its high degree of automation and ease of use [65,66,67]. These features provide valuable support in the development of tissue-engineered vascular grafts (TEVGs), especially for achieving compliance matching between the graft and native vessels, a critical parameter in coronary artery bypass grafting (CABG) failure [55,57].

**Figure A2:**
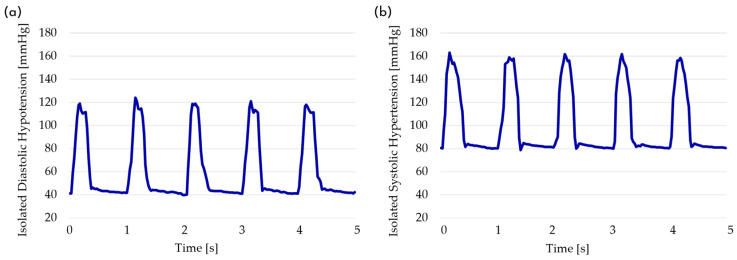
(**a**) Isolated diastolic hypotension and (**b**) isolated systolic hypertension pathological pressure profiles automatically generated using the developed platform.

Regarding coronary-like flow-induced stimulation, the platform equipped with the multi-sample chamber generates the target pressure and flow rate profiles, faithfully replicating the characteristic physiological counter-phase behavior (Figure 5a–d), and maintains the desired fluid dynamic conditions over time (Figure 5e,f). It is worth noting that there is a considerable variability in the literature regarding the exact morphology of the coronary blood flow waveform in physiological conditions. We select for our experiments a flow regime derived from a healthy human subject that lies within accepted physiological ranges [58,59,60,61,62,63]. In particular, we obtain a satisfactory flow rate variation, since most studies agree that coronary flow exhibits a pronounced diastolic-dominant peak, though the amplitude inevitably depends on myocardial mass and metabolic demand [29,30,31,63,68]. Furthermore, thanks to the feedback loop control algorithm of the pinch valve, combined with the simple and intuitive adjustment of the service compliance volume, the platform can replicate different coronary-like waveforms, offering a high degree of adaptability to a wide range of physiological and pathological conditions. As representative examples, the platform can simulate pathological pressure conditions associated with isolated diastolic hypotension and isolated systolic hypertension, as shown in Figure A2.

To contextualize the innovation and impact of our system, we evaluate the technological solutions of relevant state-of-the-art devices in the field. The system developed by Moore et al. [47] consists of a porcine aortic valve mounted within a physiological aortic sinus chamber, incorporating dynamically controlled coronary resistance to emulate realistic coronary flow. While their platform provides valuable insight into intravascular hemodynamics—particularly via particle image velocimetry (PIV) analysis in an anatomically relevant geometry—its applicability in biological studies remains limited due to its large footprint and the incompatibility of its peripheral components with standard biological workflows. Farcas et al. [48] emphasized anatomical fidelity in their incubator-compatible system, prioritizing spatial constraints over the mimicking of physiologically relevant hemodynamic stimuli. Conversely, the sophisticated system developed by Chodzyński et al. [49] offers advanced closed-loop control but lacks incubator compatibility due to its complexity and large footprint, thereby limiting the accessibility for routine biological studies and non-expert users. Piola et al. [69] proposed an ex vivo culture system designed to replicate coronary hemodynamics via time-dependent circuit resistance. Their platform was successfully used to stimulate saphenous vein grafts in a biological remodeling study. However, despite its physiologically realistic stimuli and compatibility with standard biological procedures, the system lacks automation and requires an expert operator for experimental setup and operation. In this context, the developed platform emerges as a novel solution compared with existing systems by integrating relevant technological features—such as automation, adaptability, and ease of use—into a single incubator-compatible device capable of reproducing physiologically relevant fluid-dynamic conditions in a controlled manner. Moreover, none of the advanced systems analyzed support automated 3D scaffold seeding, a key functionality for ensuring controlled and reproducible experimental conditions in vascular tissue engineering. Indeed, the developed multi-sample chamber enables longitudinal studies and is fully compatible with the previously described and validated automated rotational cell seeding protocol [53]. These features, combined with the seamless coronary-like hemodynamic stimulation, further consolidate the platform’s potential as a supportive bioengineered device for perspective investigations of critical aspects of CABG remodeling phenomena. In particular, the platform’s ability to enable longitudinal studies will be instrumental in evaluating biological responses—such as morphological changes and alterations in cellular expression—to coronary-like flow-induced stimuli over time. These investigations may focus on established markers of tissue remodeling and early intimal hyperplasia, including the increased expression of Ki67 and N-Cadherin in smooth muscle cells, as well as von Willebrand factor expression in endothelial cells [70]. From a clinically oriented perspective, the capability to perform longitudinal monitoring studies on multiple vascular samples provides a valuable framework for investigating therapeutic approaches under controlled physiologically relevant conditions. In this context, the platform can support the culture of both cell-based constructs and native vascular samples (i.e., saphenous vein segments), with the aim of correlating in vitro mechanobiological findings with clinical evidence and vascular graft outcomes. Furthermore, the system can be exploited to investigate pharmacological therapies targeting mechanisms involved in graft failure, such as inflammatory responses, smooth muscle cell proliferation, and endothelial dysfunction [71,72,73]. Indeed, the platform allows time-dependent collection of culture medium and tissue samples, enabling the evaluation of therapeutic release profiles—including, e.g., drug- and nanoparticle-based delivery—and their interaction with vascular remodeling processes over extended culture periods.

The platform presented in this work integrates automated, user-defined experimental protocols within a modular, incubator-compatible design, enabling intuitive setup and real-time monitoring of experimental parameters through the wireless graphical user interface. These features make it highly accessible to both expert and non-expert users. Thanks to its modular architecture and dedicated control algorithms, the platform also exhibits high adaptability to a broad range of experimental conditions, offering a valuable and versatile tool for supporting advanced mechanobiological investigations in vascular tissue engineering field.

## 5. Conclusions

In conclusion, the developed platform represents a valuable bioengineered tool for supporting the investigation of key biomechanical aspects relevant to the development of tissue-engineered vascular grafts (TEVGs) for coronary artery bypass grafting (CABG). Its modular design and the advanced control algorithms constitute the core technological features that enable both the compliance characterization of tubular samples and longitudinal biological studies under coronary-like fluid-dynamic conditions.

The obtained outcomes demonstrate the platform’s effectiveness to automatically generate flow-induced stimuli, ensuring a robust control over experimental conditions. In addition, the system and the defined physical procedures offer a high degree of adaptability to diverse experimental demands, further highlighting the versatility of the modular culture platform.

Overall, this platform opens new avenues for a wide range of investigations in vascular mechanobiology, particularly those focused on the interplay between compliance mismatch and biological responses to coronary-like flow-induced stimuli, with the aim of elucidating the underlying biomechanical mechanisms. In perspective, the platform has the potential to support future clinically oriented studies on native vascular samples, contributing to filling the gap between experimental mechanobiological observations and clinical evidence in vascular engineering.

## Figures and Tables

**Table 1:** Summary of the pressure–diameter data and the dynamic radial compliance of the silicone phantom under the three ISO 7198 pulsatile regimes (hypo-, normo- and hypertension). For each regime, minimum and maximum pressures ($P_{min}$, $P_{max}$), pressure variation (∆P), minimum and maximum inner diameters ($D_{P_{min}}$, $D_{P_{max}}$), and the resulting dynamic radial compliance (C) are reported as mean ± standard deviation.

	$P_{min}$ [mmHg]	$P_{max}$ [mmHg]	∆P [mmHg]	$D_{P_{min}}$ [mm]	$D_{P_{max}}$ [mm]	C [% 10^−2^ mmHg^−1^]
Hypotension	52.37 ± 0.94	92.01 ± 0.99	39.64 ± 2.24	3.215 ± 0.002	3.224 ± 0.001	0.685 ± 0.214
Normotension	79.67 ± 1.03	120.72 ± 1.60	41.06 ± 1.90	3.227 ± 0.002	3.237 ± 0.003	0.778 ± 0.225
Hypertension	110.13 ± 1.94	152.67 ± 4.65	42.53 ± 5.04	3.242 ± 0.002	3.253 ± 0.003	0.847 ± 0.248

**Table 2:** Summary of the pressure and flow rate data acquired during the long-term assessment at day 0, day 1, day 3, day 7, and day 14. For each time point, minimum and maximum pressures ($P_{min}$, $P_{max}$), pressure variation (∆P), mean flow rate ($Q_{mean}$), and flow rate variation (∆Q) are reported as mean ± standard deviation. Statistical significance is reported for each parameter (Welch’s ANOVA test).

	$P_{min}$ [mmHg]	$P_{max}$ [mmHg]	∆P [mmHg]	$Q_{mean}$ [mL/min]	∆Q [mL/min]
Day 0	78.7 ± 0.3	121.4 ± 0.5	42.8 ± 0.8	189.5 ± 5.4	168.2 ± 1.2
Day 1	77.2 ± 0.5	119.9 ± 0.7	42.7 ± 1.2	184.0 ± 4.0	163.3 ± 1.0
Day 3	78.1 ± 0.2	120.7 ± 1.2	42.6 ± 1.3	186.6 ± 2.4	167.9 ± 3.2
Day 7	79.8 ± 0.5	121.2 ± 0.8	41.4 ± 0.8	190.9 ± 3.8	167.3 ± 2.0
Day 14	79.8 ± 0.8	120.8 ± 1.3	41.2 ± 1.7	189.9 ± 2.0	163.9 ± 0.8
Statistical significance (*p*-value)	No (0.1953)	No (0.2633)	No (0.4428)	No (0.2489)	No (0.1480)

## Data Availability

The raw data supporting the conclusions of this article will be made available by the authors on request.

## Appendix A

### Appendix A.1. Graphic User Interface for Wireless Control

The platform is managed through a wireless graphical user interface (GUI) developed in LabVIEW^®^ 2023 (National Instruments, Austin, TX, USA), which allows the user to control each peripheral device in manual control (left side of the GUI, Figure A1) or set an experimental protocol by specifying the duration, the flow rate, the pressure regime, and the culture chamber rotation (right side of the GUI, Figure A1). Once the parameters are set, the connection between the GUI and the platform is no longer necessary, allowing autonomous running.

The left part of the GUI is designed to allow the operator to monitor and to directly manage the system. In “Manual control,” the user can control each peripheral device independently; manual mode is intended for testing operations or for filling and/or emptying hydraulic circuits. “Pressure sensor calibration” allows the two-point calibration of pressure transducers. The “System status” section displays platform components and operational parameters, such as temperature and humidity, which are continuously monitored to detect potential anomalies. The monitoring function offers a live plot of pressure and flow rate with filtering and data export options (central panel of the GUI, Figure A1).

The right side of the GUI is used by the operator to set the experiment parameters, allowing up to seven phases in different experimental modes. These include three main modes: stationary mode (constant flow rate and pressure), ramp mode (initial and final flow rates are set for gradual flow-induced stimuli application) or sinusoidal mode (diastolic and systolic pressures are dynamically replicated).

### Appendix A.2. Pathological Pressure Conditions

The platform is able to automatically simulate pathological pressure conditions, including isolated diastolic hypotension and isolated systolic hypertension (Figure A2) [74,75,76,77]. To obtain these pressure waveforms, the platform is configured as shown in Figure 3e and operates using the pinch-valve closure profile reported in Figure 3f.
